# Value of SPECT/CT in the diagnosis of juvenile idiopathic arthritis in young children: a retrospective case-control study

**DOI:** 10.3389/fmed.2026.1861464

**Published:** 2026-07-20

**Authors:** Junming Dong, Yihuai Xu, Yaxuan Liu, Wenhao Fan, Tong Li, Zhiming Xia, Xi Zhang, Dongjie Ren

**Affiliations:** 1 Shandong First Medical University, Jinan, Shandong, China; 2 Department of Rheumatology and Immunology, Jinan Children's Hospital, Jinan, Shandong, China; 3 Department of Nuclear Medicine, Shandong Provincial Hospital Affiliated to Shandong First Medical University, Jinan, Shandong, China; 4 Health Management Department, Jinan Huaiyin People's Hospital, Jinan, Shandong, China

**Keywords:** brightness score, juvenile idiopathic arthritis, radionuclide imaging, rheumatic, SPECT/CT

## Abstract

**Objectives:**

Juvenile idiopathic arthritis (JIA) is the most common chronic rheumatic disease in childhood, with early diagnosis remaining challenging due to its heterogeneous manifestations and complex pathogenesis. Nuclear medicine imaging, like single photon emission computed tomography (SPECT), which has proven valuable in quantifying inflammation and monitoring disease activity in adult rheumatoid arthritis (RA), may also have diagnostic potential in JIA, given the similarities in synovitis and joint damage between JIA and RA.

**Method:**

This retrospective case-control study included 29 JIA patients (aged 1–13 years) and 55 non-JIA controls (aged 1–16 years), all undergoing ^99^Tc-MDP SPECT. The standardized uptake ratio (SUR), defined as the brightness score (BS), was calculated for hip, knee, ankle, and wrist joints using the skull as a reference. Mann-Whitney U test and receiver operating characteristic (ROC) curves were used to assess differences in BS and diagnostic performance (AUC, sensitivity, specificity, and Youden's index).

**Results:**

JIA patients showed significantly higher median BS in all target joints, with the most significant differences in bilateral wrists (left wrist: median = 3.25, IQR = 1.35, *P* = 0.02; right wrist: median = 3.26, IQR = 1.5, *P* = 0.01). ROC analysis revealed limited adjunctive screening value of the wrist (left AUC = 0.780, *P* = 0.002; right AUC = 0.813, *P* = 0.000) with relatively high specificity (0.578 and 0.867) and moderate sensitivity (0.857 and 0.571), while the knee and hip had limited value (AUC ≈ 0.6).

**Conclusions:**

SPECT-based wrist brightness scoring is a potential objective imaging tool for JIA diagnosis. No significant diagnostic value was found in the hip, knee, or ankle joints.

## Introduction

Juvenile idiopathic arthritis (JIA) is a common chronic rheumatic disease in childhood and one of the most prevalent chronic conditions in children. It encompasses all forms of chronic childhood arthritis, meaning JIA is not a single disease but a collection of disorders affecting not only joints but also extra-articular structures, including the eyes, skin, and internal organs, often leading to disability or even death in children. It is defined as arthritis of unknown cause, beginning before age 16 and persisting for at least 6 weeks (1). The International League of Associations for Rheumatology (ILAR) classifies subtypes of autoimmune inflammatory diseases based on the number of affected joints, presence of systemic symptoms, and detection of rheumatoid factor (RF). These include systemic arthritis, polyarthritis, oligoarthritis, enthesitis-related arthritis, psoriatic arthritis, and undifferentiated (i.e., not fitting into any of the above categories or fitting into ≥2 categories simultaneously). Each form exhibits distinct genetic susceptibility and severity profiles (1, 2). Any arthritis not fitting these categories or attributable to >1 subtype is considered undifferentiated (3). According to 2010 European JIA incidence and prevalence statistics, JIA incidence ranged from 1.6 to 23 per 100,000, while prevalence ranged from 3.8 to 400 per 100,000. The directly standardized incidence rate was 8.2 [7.5–9.0], and the prevalence rate was 70.2 [62.9–78.1]/100,000. Prevalence and incidence rates were higher in girls than in boys (4). Early diagnosis and prompt treatment are critical for managing pediatric JIA. However, the disease presents with diverse onset patterns, disease courses, and outcomes, and its pathogenesis remains unclear, making diagnosis challenging.

Positron emission tomography (PET) and single-photon emission computed tomography (SPECT), as nuclear medicine imaging techniques, offer advantages in typical inflammatory diseases like rheumatoid arthritis. These include providing quantitative analysis results, enabling real-time monitoring of lesion tissue status, and precisely reflecting the mechanism of action of therapeutic drugs (5). Notably, JIA shares similarities with rheumatoid arthritis (RA) (6). Both represent common chronic arthritis conditions characterized by significant differences in synovial macrophage composition compared to normal synovium, accompanied by elevated systemic inflammatory mediators. They are prone to synovitis and joint tissue destruction (7, 8). Similar to adult rheumatoid arthritis (RA), radiographic scoring has been employed to assess disease severity in JIA, though this approach remains not fully established. Given the high overlap in key pathological features between JIA and RA—particularly their shared hallmarks of synovial inflammation and joint damage—we hypothesize that the quantitative assessment and dynamic monitoring capabilities demonstrated by PET and SPECT technologies in RA diagnosis and monitoring hold similar potential for evaluating JIA joint lesions. Therefore, based on the aforementioned rationale, this study conducted a retrospective analysis of accumulated SPECT imaging data from affected joints of JIA patients. The core objective is to systematically evaluate the consistency and reliability between SPECT imaging brightness characteristics and clinical diagnostic criteria. The aim is to explore and establish the potential value of radionuclide imaging brightness scoring as an auxiliary diagnostic tool for early JIA identification, disease activity assessment, and treatment efficacy monitoring by establishing an objective scoring system based on image brightness.

## Materials and methods

### Participants

**Figure 1:**
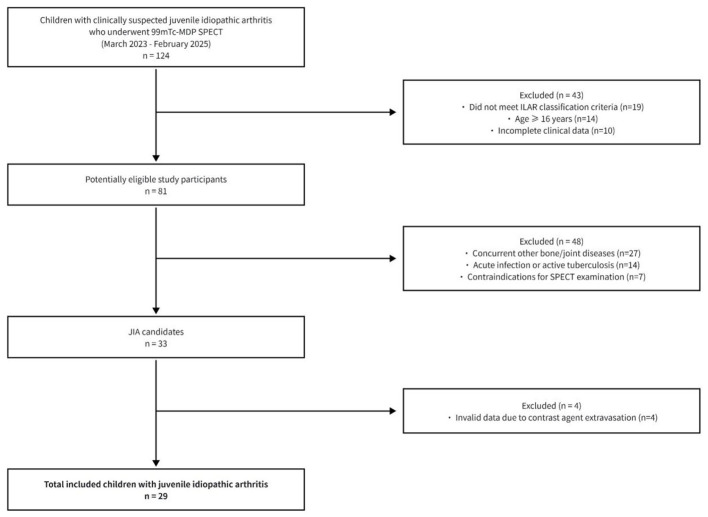
Flowchart of patient enrollment for children with juvenile idiopathic arthritis in this study.

Children clinically diagnosed with JIA at Shandong Provincial Hospital affiliated with Shandong First Medical University between March 2023 and February 2025 were enrolled (Figure 1). None of the children had received nonsteroidal anti-inflammatory drugs, glucocorticoids, or disease-modifying antirheumatic drugs prior to examination, and none of them were receiving any treatment at the time.

The control group consisted of children who underwent ^99^mTc-MDP SPECT bone scintigraphy for routine health screening, nasopharyngeal lesions, and other benign non-rheumatic and non-inflammatory conditions. Patients with malignant bone tumors, acute or chronic systemic infection, active tuberculosis, metabolic bone diseases, and any other disorders that could substantially affect bone metabolism were strictly excluded. All participants in the control group had no history of juvenile idiopathic arthritis or other rheumatic, autoimmune, and inflammatory joint diseases. We calculated the required sample size using the Schlesselman formula for matched case-control studies. Assuming a two-sided test level of α = 0.05, statistical power of 1–β = 0.80, an expected positive rate of joint SPECT brightness abnormality of 75% in JIA patients and 30% in non-JIA controls, the calculated minimum required sample size was 26 cases in each group. Our study finally enrolled 29 JIA patients and 55 controls, which met the theoretical minimum sample requirement. All subjects underwent clinical evaluation, laboratory testing, and whole-body bone scintigraphy (SPECT) using technetium-99m methylenediphosphonate (^99^mTc-MDP). Two experienced nuclear medicine physicians independently delineated all ROIs and calculated brightness scores while blinded to clinical grouping. Inter-observer reliability was assessed using Cohen's Kappa coefficient for categorical judgment and intraclass correlation coefficient (ICC) for quantitative BS values. The ICC for BS measurement was 0.892 (95%CI: 0.836–0.931, *P* < 0.001), and the Kappa value for joint abnormal judgment was 0.845, indicating excellent inter-observer agreement.

Case group inclusion criteria: (1) Diagnosis of JIA meeting the International League of Associations for Rheumatology (ILAR) classification criteria; (2) Age < 16 years; (3) Complete clinical documentation including: basic demographic information (age, gender), disease characteristics (duration of symptoms, i.e., “interval from symptom onset to SPECT examination”), and key data such as inflammatory markers [erythrocyte sedimentation rate (ESR), C-reactive protein (CRP)]. Exclusion Criteria: (1) Concurrent other bone/joint diseases (e.g., scoliosis, congenital joint deformities); (2) Presence of conditions potentially interfering with inflammatory markers (ESR, CRP), such as acute infection or active tuberculosis; (3) General contraindications for SPECT examination due to allergies, severe cardiopulmonary insufficiency, etc.

Control Group Inclusion Criteria: (1) No history of diagnosed arthritis and no diseases affecting joint development (e.g., joint dysplasia due to growth hormone deficiency); (2) Age < 16 years; (3) Complete clinical data, including basic demographic information (age, gender) and current disease diagnosis. Exclusion Criteria: (1) Presence of diseases that may interfere with inflammatory markers (ESR, CRP), such as acute infection; (2) Presence of contraindications for SPECT examination (same as case group criteria). This study was reviewed and approved by the Medical Ethics Committee of Shandong Provincial Hospital (Approval No.: SWYX: NO.2023-109).

### Bone scintigraphy imaging protocol

Bone scintigraphy and semi-quantitative analysis were conducted using the Symbia Intevo16 hybrid SPECT/CT system (Siemens Healthineers, Erlangen, Germany), which integrates a dual-head SPECT camera with a 16-slice spiral CT scanner. Bone scintigraphy was performed following intravenous injection of 15 millicuries (mCi) of technetium-99m hydroxymethylene diphosphonate (^99^mTc-HDP). All subjects underwent imaging 3 h post-injection to acquire whole-body images (“bone” phase). Patients were grouped based on expert physical examination findings into arthritis-positive and arthritis-negative cohorts (no arthritis detected by physical examination). Following a subsequent systematic evaluation by a senior pediatric rheumatologist [including but not limited to inflammatory markers, imaging, and International League of Associations for Rheumatology (ILAR) classification criteria], patients with confirmed juvenile idiopathic arthritis (JIA) were further identified from the arthritis-positive group.

Both groups underwent systematic bone imaging assessments, primarily focusing on the hip, knee, ankle, and wrist joints. Rectangular regions of interest (ROI) were delineated in the target joints and cranial regions. His process was performed independently by two nuclear medicine physicians unaware of clinical groupings. In some cases, local abnormal radioactive concentration caused by contrast agent extravasation at the intravenous injection site (wrist region) significantly interfered with quantitative analysis results in the target areas. Data from these cases were deemed invalid and excluded from the final statistical analysis. Image analysis employed a standardized method: a fixed region (area 50.24 mm^2^, selected from non-metabolically active areas) was positioned on the skull (typically metabolically stable, minimally affected by inflammation, and easily identifiable). For the sagittal plane, the region was centered on the left frontal bone, at least 0.3 cm lateral to the midline, avoiding the frontal sinus and sagittal sinus projection areas; and the left parietal bone in the posterior view, avoiding the sagittal sinus and cranial sutures. Radioactive counts from these regions serve as the baseline value (denominator). Regions of interest (ROI) were selected at each joint, centered on the joint space and defined as fixed areas (area 50.24 mm^2^). These ROI encompassed the articular surface and adjacent epiphysis (the knee joint includes distal femoral and proximal tibial epiphyses; wrist joint includes radiocarpal and intercarpal joints). The radioactivity count serves as the observed value (numerator). Brightness scoring was calculated using the standardized uptake ratio (SUR) method, with the formula:

Brightness Score (BS) = Mean Pixel Intensity of Joint ROI / Mean Pixel Intensity of Cranial Reference ROI.

All patients underwent data analysis for four joints in both anteroposterior and posteroposterior views. The final SUR value for each joint was calculated as the average of the SUR values obtained from the anteroposterior (anterior) and posteroposterior (posterior) views. Background subtraction was not performed in this study for the following reasons: minimal surrounding soft tissue around the skull minimizes interference, rendering background subtraction unnecessary; maintaining consistency between joints and skull structures is prioritized, and the blurred anatomical boundaries around pediatric joints increase the risk of manual error during background region delineation; the highly specific uptake of the radiotracer by the diseased joint makes background interference negligible, thus eliminating the need for background subtraction at the joint site.

### Statistical analysis

Continuous variables are presented as median (interquartile range, IQR). Descriptive statistical analyses were conducted for each group of data, including sample size, minimum value, 25th percentile, median, 75th percentile, maximum value, mean, standard deviation, and skewness to comprehensively evaluate data distribution characteristics. The Mann-Whitney U test was used for nonparametric comparisons to assess statistically significant differences in brightness scores between groups. The test level was set as two-tailed, with *P* < 0.05 indicating a statistically significant difference. For each examined joint (including left knee, right knee, left hip, right hip, left wrist, right wrist, left ankle, and right ankle), a receiver operating characteristic (ROC) curve was constructed, and the area under the curve (AUC) was calculated to evaluate the diagnostic performance of brightness scores for JIA. Concurrently, sensitivity, specificity, and Youden's index were calculated to determine the optimal diagnostic threshold. Furthermore, to evaluate the practical clinical value of the brightness score, precision and recall were calculated to comprehensively assess the model's classification performance. Precision measures the proportion of true positives among predicted positive results, while recall reflects the proportion of all actual positive samples correctly identified. All statistical analyses were performed using SPSS software. All statistical tests employed two-tailed significance testing, with *P* < 0.05 considered statistically significant.

## Results

**Figure 2:**
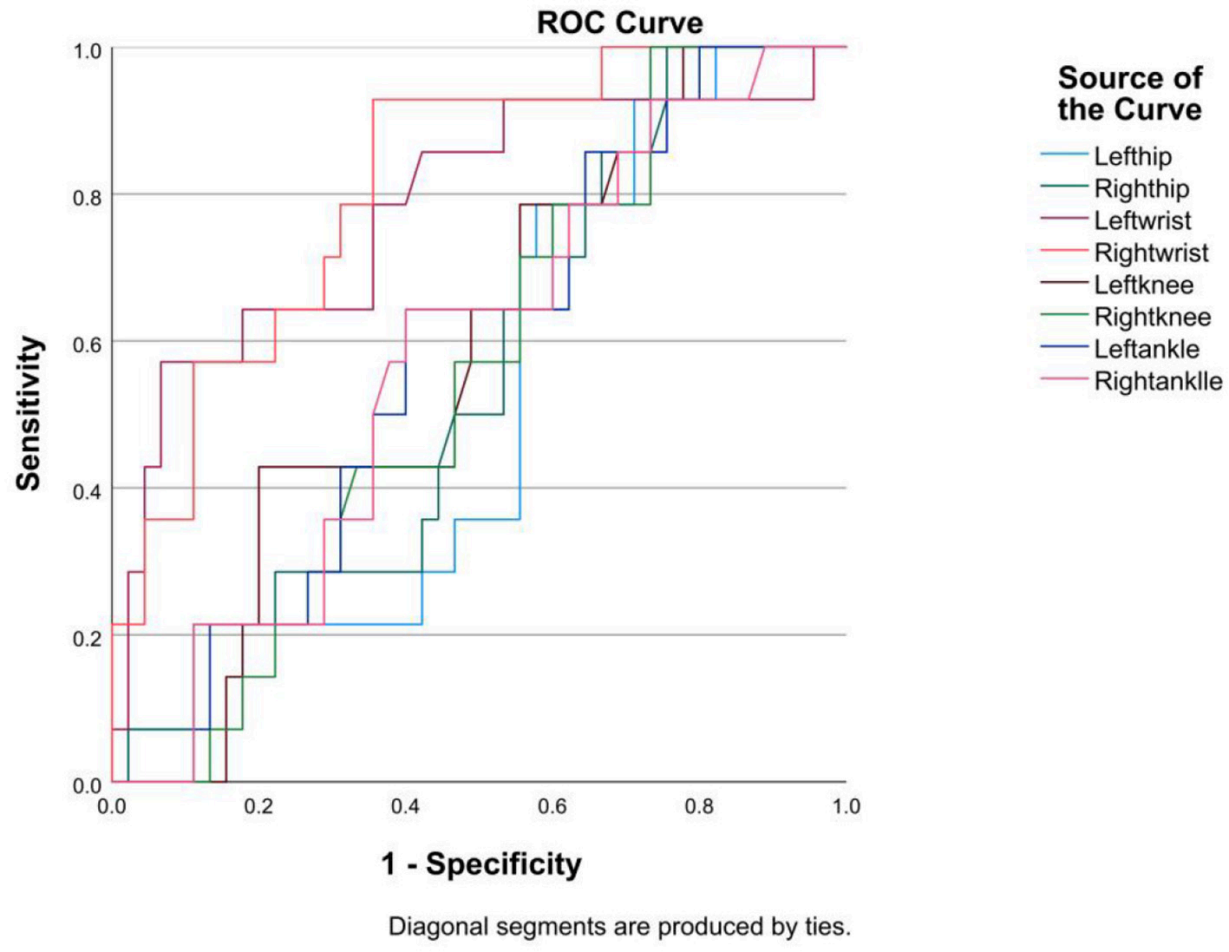
Receiver operating characteristic (ROC) curves of brightness score (BS) for diagnosing juvenile idiopathic arthritis (JIA). ROC curves were constructed for bilateral hip, wrist, knee, and ankle joints. The area under the curve (AUC) was used to evaluate the overall diagnostic performance.

1. This study included 84 pediatric patients. Among them, 55 patients who underwent radionuclide bone scans but were not diagnosed with JIA were assigned to the control group, comprising 24 males and 31 females. They ranged in age from 1 to 16 years with a mean age of 7.51 ± 4.02 years. Twenty-nine patients diagnosed with JIA based on bone scan results were assigned to the disease group, comprising 13 males and 16 females aged 1–13 years with a mean age of 8.52 ± 3.28 years. According to the ILAR classification criteria, 19 cases were systemic, two were enthesitis-related, four were oligoarticular, zero were undifferentiated, and four were polyarticular. Among these, two cases were rheumatoid factor positive and two were rheumatoid factor negative. There was no significant difference in age between the patient group and the control group (*P* = 0.101), nor was there a significant difference in gender distribution (*P* = 0.917; Table 1).
2. The chart results indicate that among all joints, JIA patients exhibited a median bone scan brightness score (BS) that was systematically higher or comparable to that of control group. The interquartile range (IQR) for both wrist joints showed minimal overlap between groups, suggesting significant differences in pathological activity (Table 2). Further analysis of BS distribution patterns revealed that the distribution of BS in the wrist and knee joints of the affected group was predominantly positively skewed, whereas the distribution in the control group was relatively symmetric, reflecting inflammatory heterogeneity within the JIA patient cohort. Additionally, the standard deviation for the knee joints in the affected group was higher than that for the wrist joints, suggesting greater heterogeneity in disease presentation in larger joints. Semi-quantitative analysis of radionuclide imaging confirmed markedly enhanced bone metabolic activity in JIA patients, particularly in the wrist joints. To quantitatively assess these differences, the Mann–Whitney U test was employed to evaluate whether brightness scores from bone scans of four joints in JIA children significantly differed from those in children with normal bone scans. Analysis of Table 2 results revealed characteristic changes in the wrist joints of JIA patients: bilateral bone scores (BS) were significantly higher than in healthy controls (left wrist: *U* = 491, *P* = 0.02; right wrist: *U* = 642, *P* = 0.01), with *P* values < 0.05 in both cases. The null hypothesis (α = 0.05) was rejected, indicating significantly higher wrist bone scan BS in the JIA group compared to healthy controls. This confirms the wrist joint as a sensitive target for abnormal bone metabolism in JIA. Although ankle joints did not reach statistical significance (*P* > 0.25), the systematic increase in median brightness scores suggests potential differences in pathological activity. No significant differences were detected in knee or hip joints (*P* > 0.30).
3. Figure 2 shows the receiver operating characteristic (ROC) curves constructed using brightness scores to evaluate diagnostic performance for JIA. Key parameters include: area under the curve (AUC), optimal cutoff value, sensitivity (Se), and specificity (Sp). ROC curves were analyzed for eight joint groups (bilateral knees, hips, wrists, and ankles). Statistical analysis revealed that the AUC values for the left wrist (AUC = 0.780, *p* = 0.002) and right wrist (AUC = 0.813, *p* < 0.001) were significantly greater than 0.5 (Table 3). This indicates that wrist brightness scores demonstrate moderate diagnostic discriminatory ability for JIA, with a statistically significant association between these scores and disease status. AUC values for other joints (e.g., hip, knee, and ankle) ranged between 0.518 and 0.583, with *p*-values exceeding 0.05. This indicates that brightness scores for these joints did not demonstrate significant diagnostic value in this study and require further validation through clinical data integration or larger sample sizes. Regarding diagnostic performance, the Youden indices for the left and right wrists were 0.435 and 0.438, respectively, higher than those of other joints. Combined with their elevated AUC values and 95% confidence intervals (left wrist: 62.8%−93.2%; right wrist: 69.3%−93.3%), this suggests more favorable and relatively stable diagnostic efficacy for the wrist joints. Regarding sensitivity (Se) and specificity (Sp), the right hip demonstrated perfect sensitivity (Se = 1.000), indicating strong patient identification capability with low risk of missed diagnosis. However, its specificity was only 0.244, suggesting a higher risk of false positives. In contrast, both the left and right wrists maintained moderate sensitivity (0.857 and 0.571, respectively) while exhibiting higher specificity (0.578 and 0.867, respectively), indicating better balance between sensitivity and specificity with improved control over false positives. Optimal cutoff values varied across joints (e.g., left wrist Cut-off = 2.39, right wrist Cut-off = 3.21), indicating distinct brightness thresholds are required for disease state differentiation in different joints. Precision and recall also showed variations across joints, such as precision of 0.86 for the left wrist vs. 0.53 for the right wrist, further reflecting heterogeneity in positive predictive ability and case identification across joints.

**Table 1:** Baseline demographic and clinical characteristics of the JIA group and control group.

Characteristics		Patient group	Control group	*P*
Age in years, mean (SD)		8.52 (3.28)	7.51 (4.02)	0.101
Gender, *n* (%)	Female	16 (55.17)	31 (56.36)	0.917
	Male	13 (44.83)	24 (43.64)	
JIA subtype, *n* (%)	Oligoarticular JIA	4 (13.79)		
	Enthesitis-related arthritis	2 (6.89)		
	Polyarticular JIA RF-	2 (6.89)		
	PolyJIA RF+	2 (6.89)		
	Systemic JIA	19 (65.51)		
	Undifferentiated JIA	0 (0)		

JIA, juvenile idiopathic arthritis; RF, rheumatoid factor. Continuous data are presented as mean ± standard deviation (SD). Categorical data are presented as *n* (%). Group comparisons were performed using Mann–Whitney U test and chi-square test. A two-sided *P* < 0.05 was considered statistically significant.

**Table 2:** Comparison of brightness score (BS) values in each joint between the JIA group and control group.

Joint	Group	Number	min	max	Mdn	M	IQR	SD	Sk	*P*	*U*
Left hip	Patient group	17	3.12	7.56	4.39	4.736	1.34	1.309	0.978	0.64	530
	Control group	58	1.82	9.55	4.68	4.567	2.22	1.552	0.537		
Right hip	Patient group	15	3.36	7.68	4.58	4.918	2.09	1.305	0.792	0.343	504
	Control group	58	1.89	10.12	4.43	4.559	2.12	1.554	0.801		
Left wrist	Patient group	14	1.22	5.21	3.25	3.176	1.35	1.007	0.096	0.002	491
	Control group	45	0.66	4.27	2.19	2.233	0.99	0.749	0.299		
Right wrist	Patient group	15	1.87	14	3.26	4.033	1.5	2.887	3.312	0.001	624
	Control group	54	0.66	4.32	2.27	2.367	1.137	0.85	0.352		
Left knee	Patient group	18	2.97	6.65	4.74	4.977	2.195	1.216	−0.032	0.193	628
	Control group	58	1.56	8.99	4.435	4.549	2.225	1.848	0.502		
Right knee	Patient group	17	3.12	7.18	4.6	4.831	1.46	1.245	0.402	0.519	544
	Control group	58	1.55	8.29	4.480	4.583	2.3175	1.812	0.237		
Left ankle	Patient group	16	1.13	5.57	2.795	2.823	1.4225	1.312	0.78	0.27	548
	Control group	58	0.4	9.37	2.145	2.596	1.925	1.72	1.618		
Right ankle	Patient group	16	0.99	5.46	2.845	2.909	1.4525	1.39	0.745	0.305	533
	Control group	57	0.38	9.75	2.2	2.673	1.88	1.761	1.608		

JIA, juvenile idiopathic arthritis; min, minimum; max, maximum; Mdn, median; M, mean; IQR, interquartile range; SD, standard deviation; Sk, skewness; U, Mann–Whitney U statistic. Data are presented as median (IQR) and mean ± SD. Between-group comparisons were performed using Mann–Whitney U test. A two-sided P < 0.05 was considered statistically significant. Variable missing data existed in both groups, with higher missing rates in the JIA group.

**Table 3:** ROC curve analysis of brightness score (BS) for diagnosing JIA in each joint.

Joint	AUC	*P*	Youden index	Se	Sp	Cut-off	95% CI	precision	recall
Left hip	0.519	0.831	0.208	0.786	0.422	3.78	35.8%−68.0%	0.76	0.28
Right hip	0.551	0.559	0.244	1	0.244	3.34	39.1%−71.1%	0.93	0.24
Left wrist	0.780	0.002	0.435	0.857	0.578	2.39	62.8%−93.2%	0.86	0.38
Right wrist	0.813	0.000	0.438	0.571	0.867	3.21	69.3%−93.3%	0.53	0.53
Left knee	0.583	0.354	0.23	0.786	0.444	3.84	42.7%−73.8%	0.83	0.31
Right knee	0.552	0.563	0.186	0.786	0.4	3.69	40.0%−70.3%	0.82	0.27
Left ankle	0.518	0.364	0.243	0.643	0.6	2.29	42.5%−73.7%	0.69	0.32
Right ankle	0.578	0.383	0.243	0.643	0.6	2.59	41.9%−73.7%	0.69	0.32

JIA, juvenile idiopathic arthritis; ROC, receiver operating characteristic; AUC, area under the curve; Se, sensitivity; Sp, specificity; CI, confidence interval. The optimal cutoff value was determined by Youden index. AUC, sensitivity, specificity, precision, and recall were calculated to evaluate diagnostic performance. A two-sided P < 0.05 was considered statistically significant.

## Discussion

**Figure 3:**
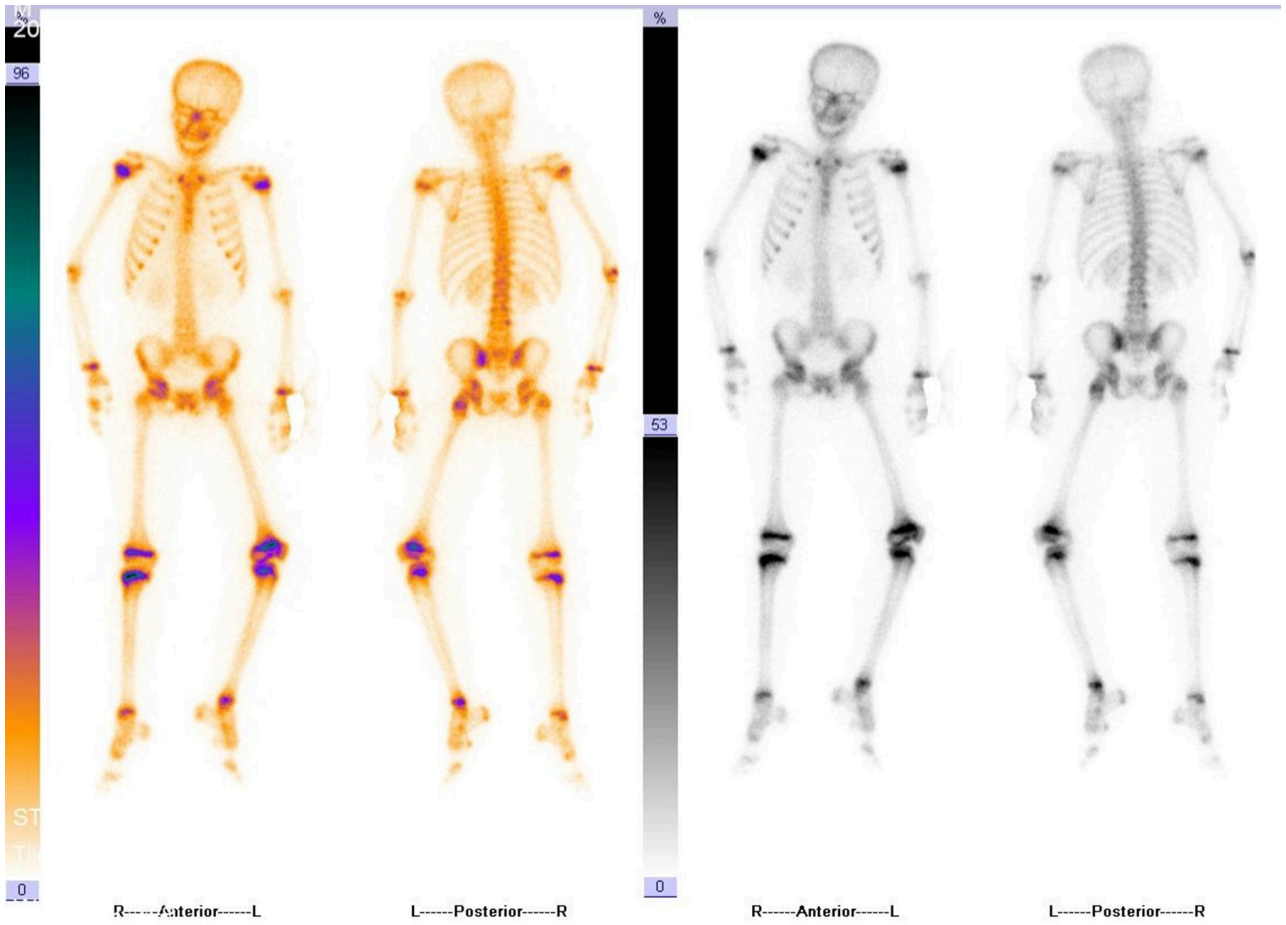
Representative bone scintigraphy images of a 12-year-old patient with systemic JIA.

This study aims to evaluate whether whole-body radionuclide imaging (SPECT) can effectively distinguish JIA from other unaffected patients. Furthermore, based on the effectiveness of this imaging modality, we sought to establish a brightness score (BS) system comparable to MRI and ultrasound, thereby developing an effective diagnostic method for JIA. Among patients presenting with clinical symptoms of arthritis, we found that, among all detection parameters, alterations in the wrist joint BS were independently and significantly associated with the JIA diagnosis at the time of the child's visit. This study included 29 JIA patients and 55 non-JIA patients. By performing whole-body SPECT examinations and calculating the SPECT imaging BS, we evaluated the diagnostic value of SPECT imaging BS in JIA. This paper first employs descriptive analysis to compare systemic differences in radionuclide uptake across joints between the patient group and the control group. JIA patients exhibit synovial hyperplasia, increased osteoclast activity, and elevated inflammatory factor activity. These conditions lead to heightened radionuclide uptake in affected joints (Figure 3), resulting in characteristic imaging alterations consistent with synovitis-driven bone metabolism changes reported in pediatric arthritis (9, 10). Building upon this theoretical framework, our SPECT imaging yielded corresponding results. Compared to the control group, the affected group exhibited significantly elevated median bone scan (BS) values across all joints, with the most pronounced increase observed in the wrist joints. This finding suggests that wrist bone uptake in JIA patients is more active than in other affected joints, seemingly consistent with the increased joint metabolism and subsequent enhanced radionuclide uptake observed in inflammatory arthritis such as rheumatoid arthritis (RA). Subsequently, the Mann-Whitney U test revealed significantly higher bilateral wrist BS in the patient group than the control group (left wrist: *U* = 491, *P* = 0.02; right wrist: *U* = 642, *P* = 0.01). This indicates the wrist joint's sensitivity to abnormal bone metabolism in JIA, with changes in its radionuclide uptake potentially serving as an indicator for JIA occurrence. Although ankle joints did not reach statistical significance (*P* > 0.25), the systematic elevation in median BS compared to the control group may suggest potential differences in pathological activity, warranting further validation studies. No significant differences in BS were detected between the knee and hip joints and the control group (all *P* > 0.30). The hip joint's deep pelvic location and surrounding tissues may compromise brightness score accuracy, suggesting its bone imaging response may be less affected by JIA. ROC curve analysis demonstrated that wrist BS exhibited limited adjunctive screening value (left wrist AUC = 0.780; right wrist AUC = 0.813), optimal specificity (left wrist: 0.578; right wrist: 0.867), and moderate sensitivity (left wrist: 0.857; right wrist: 0.571). Calculating wrist BS values and comparing them against thresholds effectively diagnoses JIA. In contrast, knee, ankle, and hip joints demonstrated lower diagnostic sensitivity (AUC ≈ 0.6), suggesting less pronounced increases in inflammatory markers due to JIA involvement in these joints. The lack of significant inter-group difference in knee and ankle joints may be attributed to active physiological growth plate uptake of ^99^mTc-MDP in children, which masks mild inflammatory metabolic changes caused by JIA; meanwhile, the deep anatomical location of the hip joint and surrounding soft tissue interference also reduce the discriminatory ability of brightness scoring. Our findings support the utility of nuclear imaging in evaluating JIA, consistent with RA studies and extendable to pediatric populations (11). The disease-activity-related joint-specific radionuclide uptake patterns observed in JIA resemble those in RA, consistent with general patterns identified in machine learning applications of bone scintigraphy for diagnosing juvenile idiopathic arthritis (12). By means of intravenous injection of ^99*m*^Tc-MDP, SPECT enables the direct quantification of bone metabolic activity in joints as a brightness score (BS) (13). MRI visualizes the structural details of joints *via* magnetic resonance signals, and X-ray imaging relies on differences in bone mineral density for image acquisition (14); however, both techniques exhibit extremely low sensitivity for detecting early synovitis and abnormal bone metabolism. Although ultrasound is radiation-free and allows real-time dynamic observation, it has limited penetration for deep joints and cannot readily quantify abnormalities at the bone metabolic level (15). In contrast, SPECT is more well-suited for the detection and quantification of early pathological features in JIA. Additionally, SPECT permits a single whole-body scan, is not affected by metallic implants (16), and requires a shorter examination time for whole-body assessment compared with other imaging modalities. While MRI is marginally more cost-effective for the evaluation of a single joint, SPECT incurs lower costs for the comprehensive assessment of multiple joints across the whole body. Notably, prior imaging studies on JIA primarily focused on ultrasound or MRI (17). No studies have directly addressed the relationship between SPECT and JIA, with occasional articles speculating its potential as a diagnostic tool for JIA, but without in-depth investigation. Our findings demonstrate that SPECT-derived bone scintigraphy, particularly at the wrist, can serve as a clinically relevant and objectively quantifiable biomarker, though it may lack specificity in early-stage disease.

This study has several limitations. First, the small sample size may limit generalizability, the present study had a relatively small single-center sample, and the distribution of JIA subtypes was unbalanced, with systemic JIA accounting for 65.51% of all cases. This uneven subtype composition may limit the generalizability of the findings to the full spectrum of JIA subtypes. Further multicenter studies with larger sample sizes and balanced subtype recruitment are needed to verify our conclusions. Second, as a retrospective study, selection bias may exist since controls were non-JIA patients undergoing SPECT for other reasons, potentially introducing unavoidable confounding factors. Third, we employed ^99^mTc-MDP, which primarily reflects osteoblast activity rather than inflammation-specific tracers, potentially affecting assessment accuracy. Moreover, SPECT/CT involves ionizing radiation, which limits its routine application in pediatric patients, especially young children. Therefore, although quantitative bone SPECT/CT shows certain auxiliary value, it should be considered only for selective and clinically complex cases rather than routine screening. Fourth, the skull was selected as the reference region for BS normalization. In children aged 1–16 years, the calvarial bone exhibits relatively stable physiological bone metabolic activity with less susceptibility to peripheral joint inflammation and local lesion interference compared with limb joints. Moreover, the skull ROI was placed in the frontal and parietal bone regions avoiding cranial sutures, frontal sinus and vascular sinus projection areas, which further reduced the influence of developmental bone turnover. Nevertheless, subtle physiological bone metabolism differences caused by rapid growth in young children cannot be completely eliminated, which is a potential limitation of skull reference normalization in pediatric SPECT quantitative analysis. Future studies should include large-scale prospective research recruiting larger, subtype-stratified cohorts to validate wrist bone score as a diagnostic and prognostic marker across JIA subtypes. Tracer optimization should be pursued, exploring inflammation-specific radiotracers (e.g., ^18^F-FDG) to enhance sensitivity for detecting JIA synovitis.

## Conclusion

Our study suggests that SPECT-derived wrist brightness scores represent a promising objective adjunctive tool for the auxiliary diagnosis of JIA, with potential applications in early detection and disease activity assessment. These findings provide preliminary support for the potential integration of nuclear imaging into pediatric rheumatology practice as a complement to conventional assessments to improve diagnostic accuracy and patient management.

## Data Availability

The original contributions presented in the study are included in the article/supplementary material, further inquiries can be directed to the corresponding authors.
